# A drought stress transcriptome profiling as the first genomic resource for white teak - Gamhar - (*Gmelina arborea* Roxb) and related species

**DOI:** 10.1186/1753-6561-5-S7-P178

**Published:** 2011-09-13

**Authors:** Carolina Rosero, Xavier Argout, Manuel Ruiz, Wilson Teran

**Affiliations:** 1 Unidad de Biotecnología Vegetal, Universidad Javeriana, Bogotá, Colombia; 2 UMR DAP, CIRAD – Montpellier, France

## Background

Gmelina (*Gmelina arborea* Roxb), also known as *white teak* is a tropical deciduous tree native from moist tropical forests of Asia. It has been successfully introduced in equatorial Africa, as well as central and south America. Gmelina is known for its very fast growing rate, its intrinsic disease, fire and drought tolerance, as well as the quality of its wood which is suitable for different types of uses such as paper pulping, plywood or particle board industry, furniture and light constructions. In addition, gmelina is considered a pioneer plant species capable of rapidly colonizing eroded or low nutritional quality lands, which makes it interesting for reforestation or landscape restoration programs. Despite its ecological and increasing economic importance, very little is known about the biology of this species and its remarkable field behavior such as drought tolerance, at the genetic, molecular and biochemical levels.

Genomic tools have recently increased the numbers and volume of genomic resources for several crop plants and trees and have contributed to enlarge our knowledge on basic aspects of plant biology, population dynamics or phylogeny; furthermore, they represent valuable sources of candidate genes and new molecular markers to assist breeding programs. Biological sequences reported to date in public databases and belonging to Gmelina do not exceed 20 entries: this very narrow availability of genetic information is the main bottleneck to initiate molecular breeding programs in *Gmelina arborea*.

We report here the first genomic resource for this tropical tree based on an RNAseq transcriptome profiling approach.

## Methods

Two subtracted cDNA libraries were constructed from both leaf and root tissue of gmelina seedlings submitted to a water deficit stress. Subtracted cDNA sequencing was completed using 454 GS FLX Titanium sequencing. After sequence trimming, de novo assembly and clustering were completed using TGICL assembler and contigs were functionally annotated using BlastX against high quality curated protein reference databases. Both Gene Ontology (GO) terms and KEGG metabolic pathways assignments were used to further characterize transcripts. SSR motives were identified using MISA. Except for the KEGG pathway assignments, all the sequence analysis was compiled in the previously developed bioinformatic pipeline ESTTIK [http://esttik.cirad.fr/].

## Results

A preliminary collection of 10.528 contigs and 10.661 singletons, enriched in drought related transcripts, was obtained from both root and leaf subtracted libraries. Contig sequence sizes ranged from 97 to 2.187 bp with an average of 456 bp and a mean coverage depth of 38-fold. Functional annotation was completed only for contigs: up to 65% of these assembled sequences had significant Blast hits and about half of all assembled contigs could be assigned to one or more GO terms (Table 1).

**Table 1:** Summary of Gmelina arborea transcript sequencing and functional annotation

		LIBRARY	Leaves	Roots	TOTAL
ASSEMBLY		Input sequences	401.181	87.078	488.259
		Clean sequences	340.654 (85%)	74.480 (86%)	415.134 (85%)
		Singleton	6.045	4.616	10.661
		**Contigs**	**5.696**	**4.832**	**10.528**
		Size range (average) (bp)	100-1731 (413)	97-2197 (499)	97-2197 (456)
		Depth coverage (fold)	59	14	38

Annotation	I	**Blast Hit**	**4.185 (73%)**	**2.623 (54%)**	**6.808 (65%)**
		No Blast hi	1.511	2.209	3.720
	II	**GO annotated contigs**	**3.274 (57%)**	**1.743 (36%)**	**5.017 (48%)**
		GO terms	20.965	10.474	31.439
		Contigs with Enzime Codes	1.252	509	1.761
	III	**KEGG pathway assigned contigs**	**738 (13%)**	**292 (6%)**	**1.030 (10%)**

^a^ Percentages are related to total number of contigs

Top hit species for homology based annotations of gmelina unigenes were: *Arabidopsis thaliana* (20-27%), *Vitis vinifera* (10-14%), *Populus trichocarpa* (4-7 %) and *Ricinus communis* (4-7%). Among the different biological processes, cellular and metabolic processes, biological regulation, localization and response to stimulus were the most highly represented GO term categories, involving several genes related to drought or general stress, transport, transcription regulation and signal transduction, as expected. Analysis of KEGG metabolic pathway assignments revealed that our gene catalog covers all major plant metabolic pathways, with a certain dominance of enzymes of the carbohydrate, amino acid and energy metabolism in leaves, indicative that those pathways, seemingly impaired in response to water deficit stress, tend to recover by means of an active transcriptional rate (Table 2).

**Table 2:** Principal KEGG metabolic pathway assignments

Metabolic pathway	Leaves	%	Roots	%	Total	%
**Carbohydrate**	348	20.5	99	15.0	447	18.9
**Energy**	362	21.3	87	13.2	449	19.0
**Lipid**	119	7.0	106	16.1	225	9.5
**Nucleotide**	60	3.5	45	6.8	105	4.4
**Amino Acid**	299	17.6	118	17.9	417	17.7
**Other Amino Acids**	67	3.9	28	4.2	95	4.0
**Glycan Biosynthesis**	13	0.8	19	2.9	32	1.4
**Cofactors and Vitamins**	124	7.3	33	5.0	157	6.7
**Terpenoids and Polyketides**	66	3.9	19	2.9	85	3.6
**Other Secondary Metabolites**	128	7.5	46	7.0	174	7.4
**Other**	114	6.7	60	9.1	174	7.4

**Total Assignments**	**1700**		**660**		**2360**	

The metabolic response in roots was slightly different with dominance of enzymes related to lipid, amino acid and carbohydrate metabolism, supporting important membrane and osmotic adjustment mechanisms in these tissues. Importantly, our gene catalog comprises many genes encoding proteins involved in signal perception and transduction, effector proteins as well as proteins with regulatory functions, allowing to cover the whole molecular response to drought stress. On the other hand the presence of several unigenes with no blast hit or with homology to unknown hypothetical proteins opens the possibility to uncover either previously unknown or less characterized protein functions related to drought stress.

Finally, we also identified microsatellite motifs within 428 unigenes, from which, 255 primer pairs have been designed for further experimental validation as functional SSR markers.

## Conclusions

In this survey, we present the first unigene resource of this economically important tropical timber for which almost no prior genetic information existed. We identified drought stress related genes in different functional categories ranging from membrane bound sensor proteins, signal transduction proteins, transcription factors to metabolic or effector proteins. The SSR motifs found are good candidates for drought-related functional marker development in this species. Altogether, the results represent a first contribution to a better knowledge of the biology of *white teak* as well as the molecular mechanisms underlying its drought tolerance, which is essential to further encourage breeding program developments.

